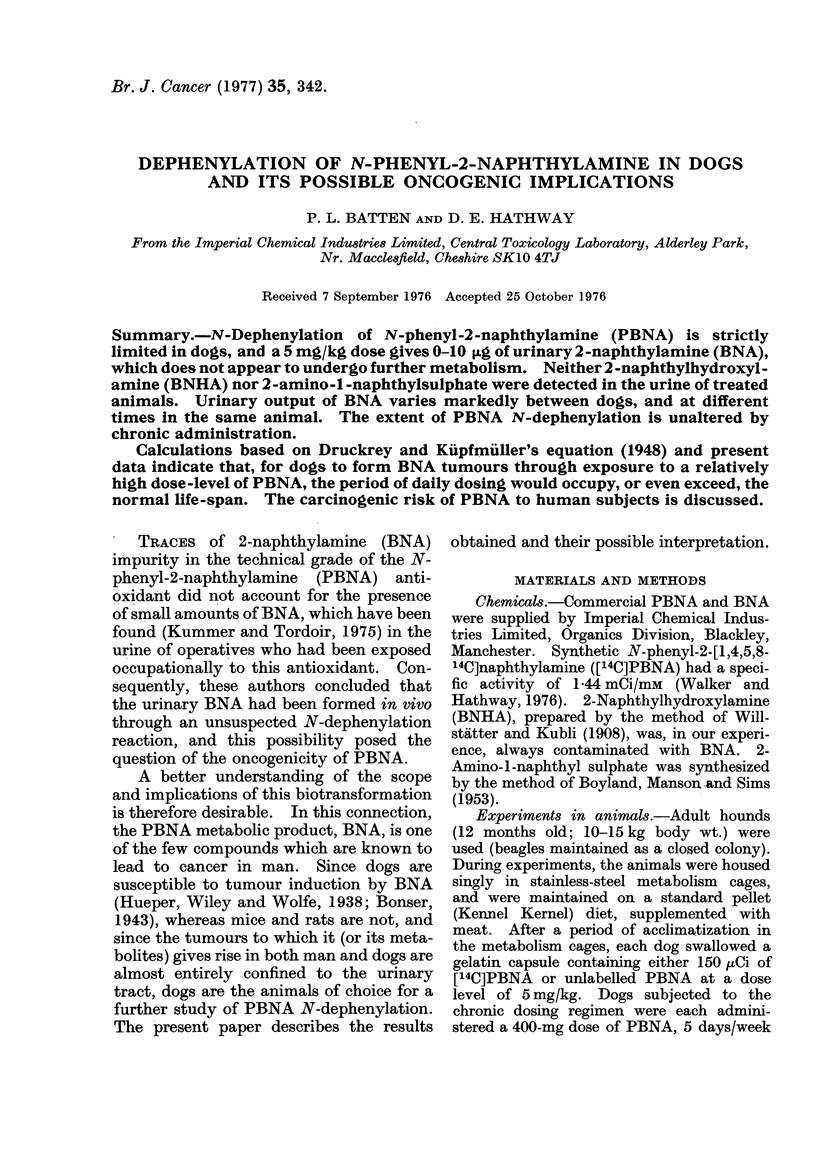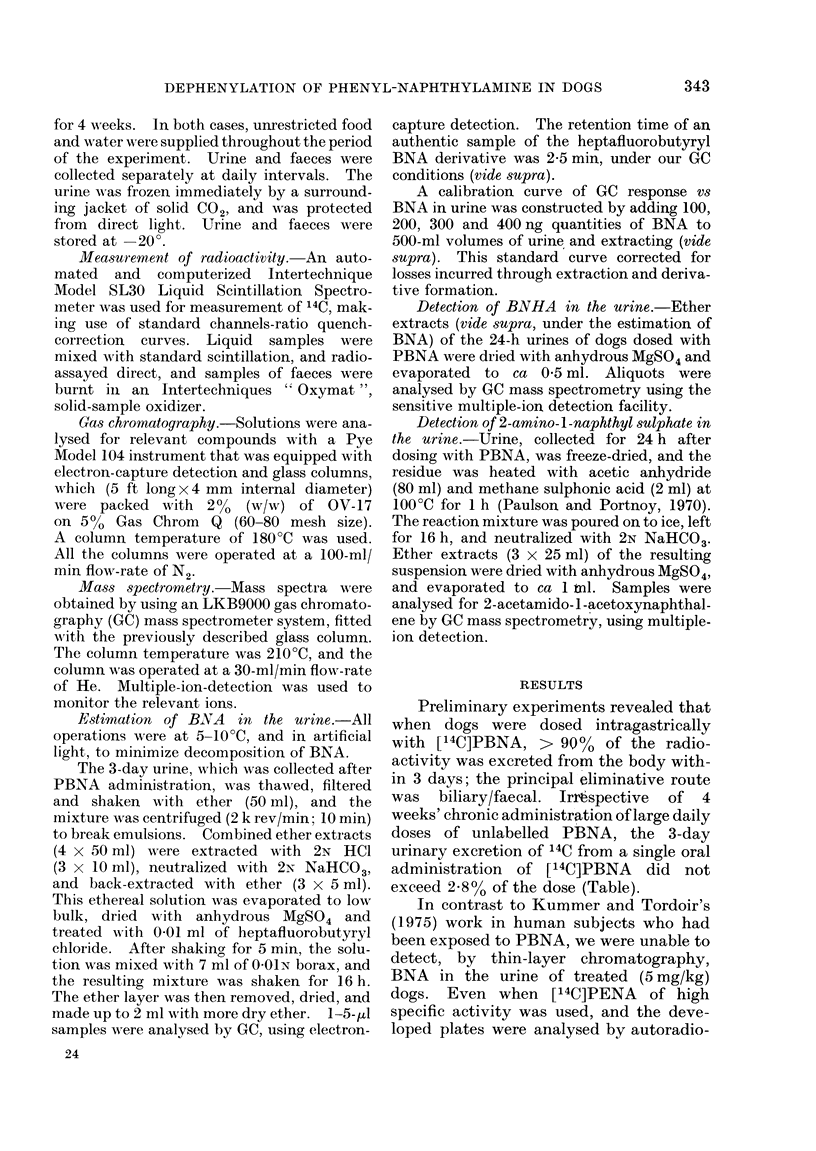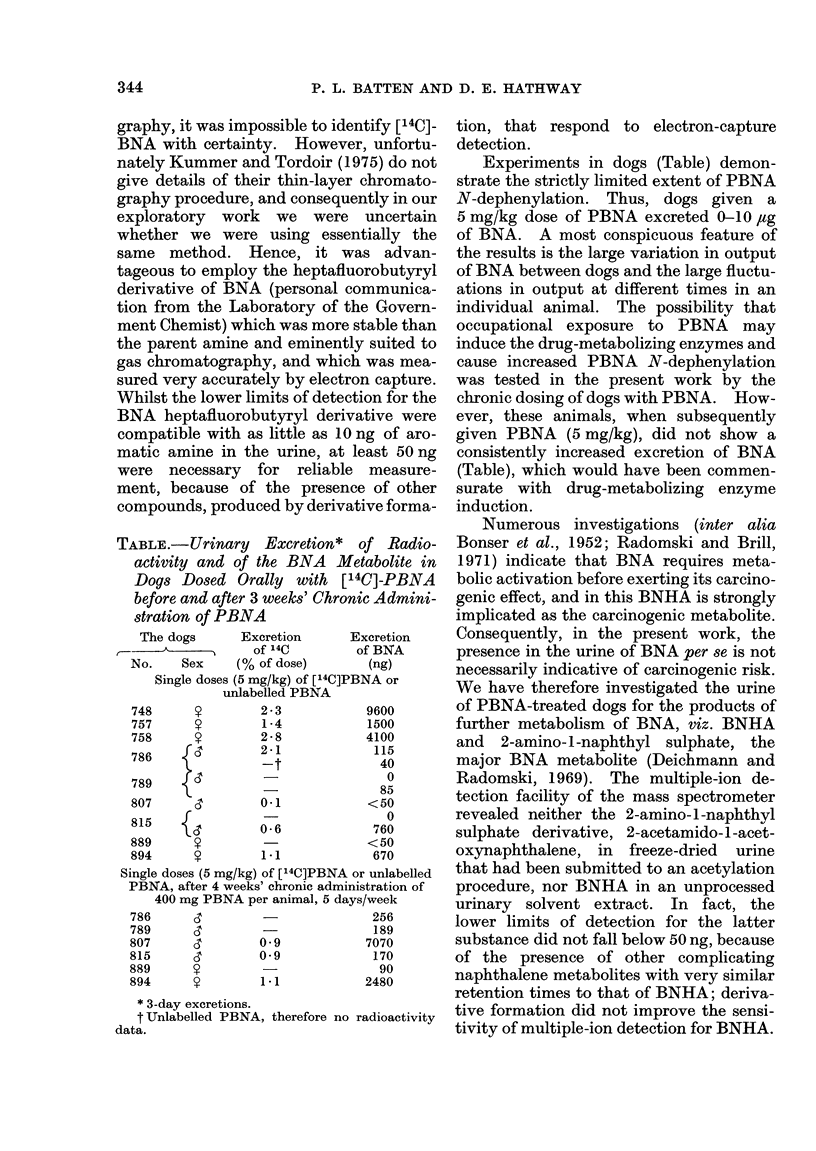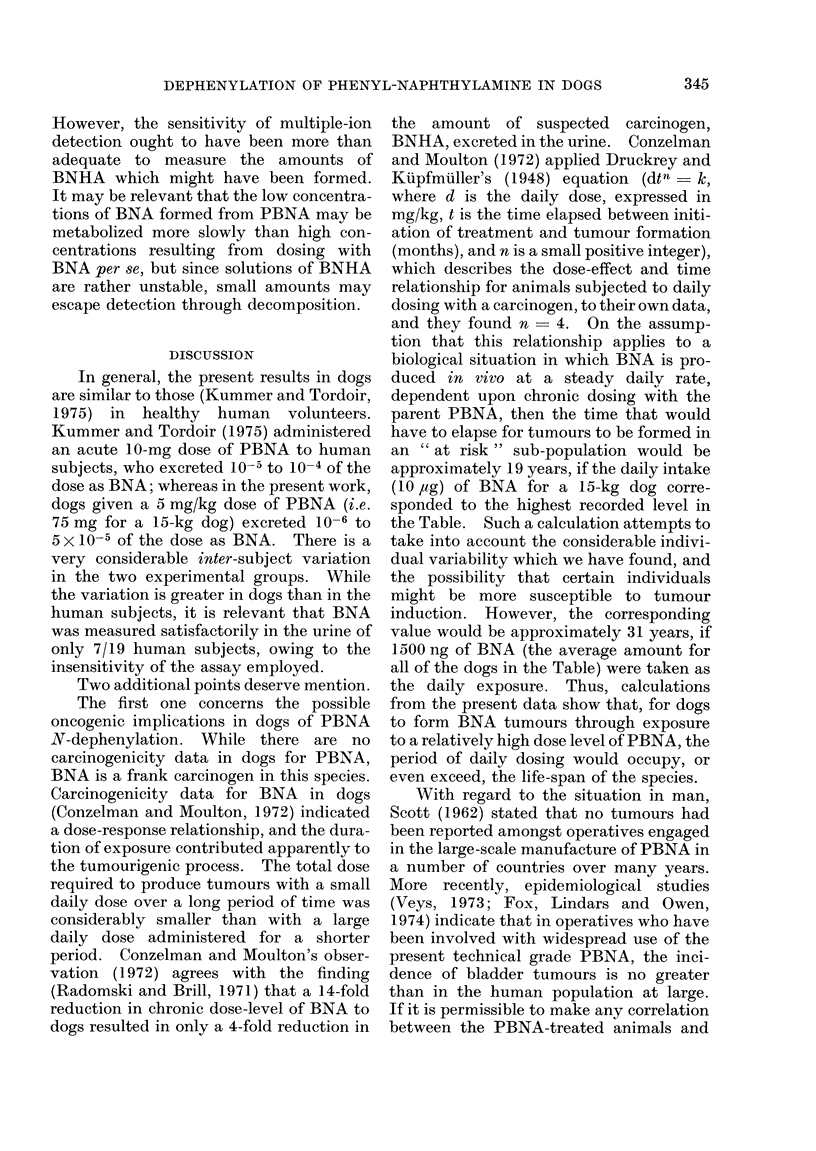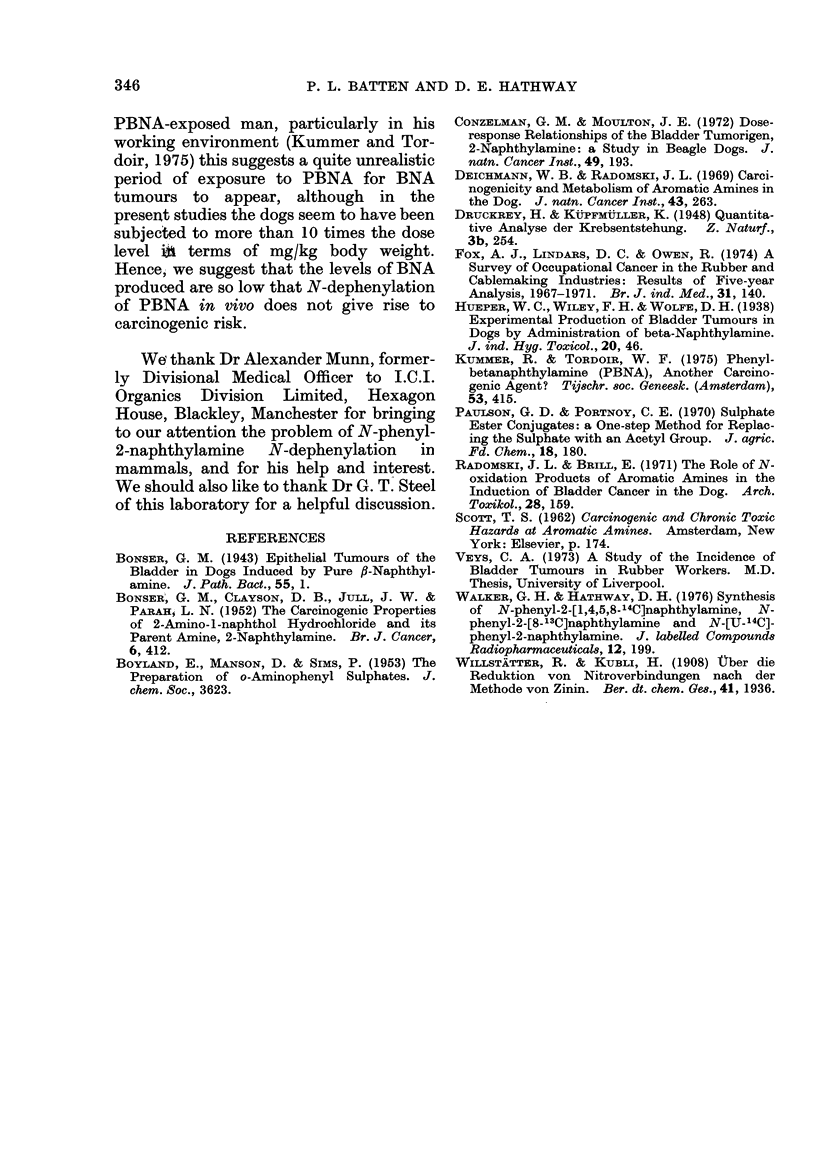# Dephenylation of N-phenyl-2-naphthylamine in dogs and its possible oncogenic implications.

**DOI:** 10.1038/bjc.1977.48

**Published:** 1977-03

**Authors:** P. L. Batten, D. E. Hathway

## Abstract

N-Dephenylation of N-phenyl-2-naphthylamine (PBNA) is strictly limited in dogs, and a 5 mg/kg dose gives 0-10 microng of urinary 2-naphthylamine (BNA), which does not appear to undergo further metabolism. Neither 2-naphthylhydroxylamine (BNHA) nor 2-amino-1-naphthylsulphate were detected in the urine of treated animals. Urinary output of BNA varies markedly between dogs, and at different times in the same animal. The extent of PBNA N-dephenylation is unaltered by chronic administration. Calculations based on Druckery and Küpfmüller's equation (1948) and present data indicate that, for dogs to form BNA tumours through exposure to a relatively high dose-level of PBNA, the period of daily dosing would occupy, or even exceed, the normal life-span. The carcinogenic risk of PBNA to human subjects is discussed.


*Br. J. Cancer* (1977) **35**, 342.

### DEPHENYLATION OF *N*-PHENYL-2-NAPHTHYLAMINE IN DOGS AND ITS POSSIBLE ONCOGENIC IMPLICATIONS

P. L. BATTEN AND D. E. HATHWAY

*From the Imperial Chemical Industries Limited, Central Toxicology Laboratory, Alderley Park, Nr. Macclesfield, Cheshire SK10 4TJ*

Received 7 September 1976 Accepted 25 October 1976

**Summary.—*N*-Dephenylation of *N*-phenyl-2-naphthylamine (PBNA) is strictly limited in dogs, and a 5 mg/kg dose gives 0–10 μg of urinary 2-naphthylamine (BNA), which does not appear to undergo further metabolism. Neither 2-naphthylhydroxylamine (BNHA) nor 2-amino-1-naphthylsulphate were detected in the urine of treated animals. Urinary output of BNA varies markedly between dogs, and at different times in the same animal. The extent of PBNA *N*-dephenylation is unaltered by chronic administration.**

**Calculations based on Druckrey and Küpfmüller's equation (1948) and present data indicate that, for dogs to form BNA tumours through exposure to a relatively high dose-level of PBNA, the period of daily dosing would occupy, or even exceed, the normal life-span. The carcinogenic risk of PBNA to human subjects is discussed.**

TRACES of 2-naphthylamine (BNA) impurity in the technical grade of the *N*-phenyl-2-naphthylamine (PBNA) antioxidant did not account for the presence of small amounts of BNA, which have been found (Kummer and Tordoir, 1975) in the urine of operatives who had been exposed occupationally to this antioxidant. Consequently, these authors concluded that the urinary BNA had been formed *in vivo* through an unsuspected *N*-dephenylation reaction, and this possibility posed the question of the oncogenicity of PBNA.

A better understanding of the scope and implications of this biotransformation is therefore desirable. In this connection, the PBNA metabolic product, BNA, is one of the few compounds which are known to lead to cancer in man. Since dogs are susceptible to tumour induction by BNA (Hueper, Wiley and Wolfe, 1938; Bonser, 1943), whereas mice and rats are not, and since the tumours to which it (or its metabolites) gives rise in both man and dogs are almost entirely confined to the urinary tract, dogs are the animals of choice for a further study of PBNA *N*-dephenylation. The present paper describes the results obtained and their possible interpretation.

### MATERIALS AND METHODS

*Chemicals.*—Commercial PBNA and BNA were supplied by Imperial Chemical Industries Limited, Organics Division, Blackley, Manchester. Synthetic *N*-phenyl-2-[1,4,5,8-$^{14}$C]naphthylamine ([$^{14}$C]PBNA) had a specific activity of 1·44 mCi/mM (Walker and Hathway, 1976). 2-Naphthylhydroxylamine (BNHA), prepared by the method of Willstätter and Kubli (1908), was, in our experience, always contaminated with BNA. 2-Amino-1-naphthyl sulphate was synthesized by the method of Boyland, Manson and Sims (1953).

*Experiments in animals.*—Adult hounds (12 months old; 10–15 kg body wt.) were used (beagles maintained as a closed colony). During experiments, the animals were housed singly in stainless-steel metabolism cages, and were maintained on a standard pellet (Kennel Kernel) diet, supplemented with meat. After a period of acclimatization in the metabolism cages, each dog swallowed a gelatin capsule containing either 150 μCi of [$^{14}$C]PBNA or unlabelled PBNA at a dose level of 5 mg/kg. Dogs subjected to the chronic dosing regimen were each administered a 400-mg dose of PBNA, 5 days/week for 4 weeks. In both cases, unrestricted food and water were supplied throughout the period of the experiment. Urine and faeces were collected separately at daily intervals. The urine was frozen immediately by a surrounding jacket of solid $CO_2$, and was protected from direct light. Urine and faeces were stored at $-20°$.

*Measurement of radioactivity.*—An automated and computerized Intertechnique Model SL30 Liquid Scintillation Spectrometer was used for measurement of $^{14}C$, making use of standard channels-ratio quench-correction curves. Liquid samples were mixed with standard scintillation, and radioassayed direct, and samples of faeces were burnt in an Intertechniques " Oxymat ", solid-sample oxidizer.

*Gas chromatography.*—Solutions were analysed for relevant compounds with a Pye Model 104 instrument that was equipped with electron-capture detection and glass columns, which (5 ft long × 4 mm internal diameter) were packed with 2% (w/w) of OV-17 on 5% Gas Chrom Q (60–80 mesh size). A column temperature of 180°C was used. All the columns were operated at a 100-ml/min flow-rate of $N_2$.

*Mass spectrometry.*—Mass spectra were obtained by using an LKB9000 gas chromatography (GC) mass spectrometer system, fitted with the previously described glass column. The column temperature was 210°C, and the column was operated at a 30-ml/min flow-rate of He. Multiple-ion-detection was used to monitor the relevant ions.

*Estimation of BNA in the urine.*—All operations were at 5–10°C, and in artificial light, to minimize decomposition of BNA.

The 3-day urine, which was collected after PBNA administration, was thawed, filtered and shaken with ether (50 ml), and the mixture was centrifuged (2 k rev/min; 10 min) to break emulsions. Combined ether extracts (4 × 50 ml) were extracted with 2N HCl (3 × 10 ml), neutralized with 2N $NaHCO_3$, and back-extracted with ether (3 × 5 ml). This ethereal solution was evaporated to low bulk, dried with anhydrous $MgSO_4$ and treated with 0·01 ml of heptafluorobutyryl chloride. After shaking for 5 min, the solution was mixed with 7 ml of 0·01N borax, and the resulting mixture was shaken for 16 h. The ether layer was then removed, dried, and made up to 2 ml with more dry ether. 1–5-μl samples were analysed by GC, using electron-capture detection. The retention time of an authentic sample of the heptafluorobutyryl BNA derivative was 2·5 min, under our GC conditions (*vide supra*).

A calibration curve of GC response *vs* BNA in urine was constructed by adding 100, 200, 300 and 400 ng quantities of BNA to 500-ml volumes of urine and extracting (*vide supra*). This standard curve corrected for losses incurred through extraction and derivative formation.

*Detection of BNHA in the urine.*—Ether extracts (*vide supra*, under the estimation of BNA) of the 24-h urines of dogs dosed with PBNA were dried with anhydrous $MgSO_4$ and evaporated to *ca* 0·5 ml. Aliquots were analysed by GC mass spectrometry using the sensitive multiple-ion detection facility.

*Detection of* 2-*amino*-1-*naphthyl sulphate in the urine.*—Urine, collected for 24 h after dosing with PBNA, was freeze-dried, and the residue was heated with acetic anhydride (80 ml) and methane sulphonic acid (2 ml) at 100°C for 1 h (Paulson and Portnoy, 1970). The reaction mixture was poured on to ice, left for 16 h, and neutralized with 2N $NaHCO_3$. Ether extracts (3 × 25 ml) of the resulting suspension were dried with anhydrous $MgSO_4$, and evaporated to *ca* 1 ml. Samples were analysed for 2-acetamido-1-acetoxynaphthalene by GC mass spectrometry, using multiple-ion detection.

### RESULTS

Preliminary experiments revealed that when dogs were dosed intragastrically with [$^{14}C$]PBNA, > 90% of the radioactivity was excreted from the body within 3 days; the principal eliminative route was biliary/faecal. Irrespective of 4 weeks' chronic administration of large daily doses of unlabelled PBNA, the 3-day urinary excretion of $^{14}C$ from a single oral administration of [$^{14}C$]PBNA did not exceed 2·8% of the dose (Table).

In contrast to Kummer and Tordoir's (1975) work in human subjects who had been exposed to PBNA, we were unable to detect, by thin-layer chromatography, BNA in the urine of treated (5 mg/kg) dogs. Even when [$^{14}C$]PENA of high specific activity was used, and the developed plates were analysed by autoradiography, it was impossible to identify [$^{14}$C]-BNA with certainty. However, unfortunately Kummer and Tordoir (1975) do not give details of their thin-layer chromatography procedure, and consequently in our exploratory work we were uncertain whether we were using essentially the same method. Hence, it was advantageous to employ the heptafluorobutyryl derivative of BNA (personal communication from the Laboratory of the Government Chemist) which was more stable than the parent amine and eminently suited to gas chromatography, and which was measured very accurately by electron capture. Whilst the lower limits of detection for the BNA heptafluorobutyryl derivative were compatible with as little as 10 ng of aromatic amine in the urine, at least 50 ng were necessary for reliable measurement, because of the presence of other compounds, produced by derivative formation, that respond to electron-capture detection.

TABLE.—*Urinary Excretion* of Radioactivity and of the BNA Metabolite in Dogs Dosed Orally with* [$^{14}$*C*]*-PBNA before and after* 3 *weeks' Chronic Administration of PBNA*

| The dogs | | Excretion of $^{14}$C | Excretion of BNA |
| --- | --- | --- | --- |
| No. | Sex | (% of dose) | (ng) |
| Single doses (5 mg/kg) of [$^{14}$C]PBNA or unlabelled PBNA | | | |
| 748 | ♀ | 2·3 | 9600 |
| 757 | ♀ | 1·4 | 1500 |
| 758 | ♀ | 2·8 | 4100 |
| 786 | ♂ | 2·1 | 115 |
| | | —† | 40 |
| 789 | ♂ | — | 0 |
| | | — | 85 |
| 807 | ♂ | 0·1 | <50 |
| 815 | | — | 0 |
| | ♂ | 0·6 | 760 |
| 889 | ♀ | — | <50 |
| 894 | ♀ | 1·1 | 670 |
| Single doses (5 mg/kg) of [$^{14}$C]PBNA or unlabelled PBNA, after 4 weeks' chronic administration of 400 mg PBNA per animal, 5 days/week | | | |
| 786 | ♂ | — | 256 |
| 789 | ♂ | — | 189 |
| 807 | ♂ | 0·9 | 7070 |
| 815 | ♂ | 0·9 | 170 |
| 889 | ♀ | — | 90 |
| 894 | ♀ | 1·1 | 2480 |

* 3-day excretions.
† Unlabelled PBNA, therefore no radioactivity data.

Experiments in dogs (Table) demonstrate the strictly limited extent of PBNA *N*-dephenylation. Thus, dogs given a 5 mg/kg dose of PBNA excreted 0–10 $\mu$g of BNA. A most conspicuous feature of the results is the large variation in output of BNA between dogs and the large fluctuations in output at different times in an individual animal. The possibility that occupational exposure to PBNA may induce the drug-metabolizing enzymes and cause increased PBNA *N*-dephenylation was tested in the present work by the chronic dosing of dogs with PBNA. However, these animals, when subsequently given PBNA (5 mg/kg), did not show a consistently increased excretion of BNA (Table), which would have been commensurate with drug-metabolizing enzyme induction.

Numerous investigations (*inter alia* Bonser *et al.*, 1952; Radomski and Brill, 1971) indicate that BNA requires metabolic activation before exerting its carcinogenic effect, and in this BNHA is strongly implicated as the carcinogenic metabolite. Consequently, in the present work, the presence in the urine of BNA *per se* is not necessarily indicative of carcinogenic risk. We have therefore investigated the urine of PBNA-treated dogs for the products of further metabolism of BNA, *viz.* BNHA and 2-amino-1-naphthyl sulphate, the major BNA metabolite (Deichmann and Radomski, 1969). The multiple-ion detection facility of the mass spectrometer revealed neither the 2-amino-1-naphthyl sulphate derivative, 2-acetamido-1-acetoxynaphthalene, in freeze-dried urine that had been submitted to an acetylation procedure, nor BNHA in an unprocessed urinary solvent extract. In fact, the lower limits of detection for the latter substance did not fall below 50 ng, because of the presence of other complicating naphthalene metabolites with very similar retention times to that of BNHA; derivative formation did not improve the sensitivity of multiple-ion detection for BNHA.

However, the sensitivity of multiple-ion detection ought to have been more than adequate to measure the amounts of BNHA which might have been formed. It may be relevant that the low concentrations of BNA formed from PBNA may be metabolized more slowly than high concentrations resulting from dosing with BNA *per se*, but since solutions of BNHA are rather unstable, small amounts may escape detection through decomposition.

### DISCUSSION

In general, the present results in dogs are similar to those (Kummer and Tordoir, 1975) in healthy human volunteers. Kummer and Tordoir (1975) administered an acute 10-mg dose of PBNA to human subjects, who excreted $10^{-5}$ to $10^{-4}$ of the dose as BNA; whereas in the present work, dogs given a 5 mg/kg dose of PBNA (*i.e.* 75 mg for a 15-kg dog) excreted $10^{-6}$ to $5\times10^{-5}$ of the dose as BNA. There is a very considerable *inter*-subject variation in the two experimental groups. While the variation is greater in dogs than in the human subjects, it is relevant that BNA was measured satisfactorily in the urine of only 7/19 human subjects, owing to the insensitivity of the assay employed.

Two additional points deserve mention.

The first one concerns the possible oncogenic implications in dogs of PBNA *N*-dephenylation. While there are no carcinogenicity data in dogs for PBNA, BNA is a frank carcinogen in this species. Carcinogenicity data for BNA in dogs (Conzelman and Moulton, 1972) indicated a dose-response relationship, and the duration of exposure contributed apparently to the tumourigenic process. The total dose required to produce tumours with a small daily dose over a long period of time was considerably smaller than with a large daily dose administered for a shorter period. Conzelman and Moulton's observation (1972) agrees with the finding (Radomski and Brill, 1971) that a 14-fold reduction in chronic dose-level of BNA to dogs resulted in only a 4-fold reduction in the amount of suspected carcinogen, BNHA, excreted in the urine. Conzelman and Moulton (1972) applied Druckrey and Küpfmüller's (1948) equation ($dt^n = k$, where $d$ is the daily dose, expressed in mg/kg, $t$ is the time elapsed between initiation of treatment and tumour formation (months), and $n$ is a small positive integer), which describes the dose-effect and time relationship for animals subjected to daily dosing with a carcinogen, to their own data, and they found $n = 4$. On the assumption that this relationship applies to a biological situation in which BNA is produced *in vivo* at a steady daily rate, dependent upon chronic dosing with the parent PBNA, then the time that would have to elapse for tumours to be formed in an " at risk " sub-population would be approximately 19 years, if the daily intake (10 $\mu$g) of BNA for a 15-kg dog corresponded to the highest recorded level in the Table. Such a calculation attempts to take into account the considerable individual variability which we have found, and the possibility that certain individuals might be more susceptible to tumour induction. However, the corresponding value would be approximately 31 years, if 1500 ng of BNA (the average amount for all of the dogs in the Table) were taken as the daily exposure. Thus, calculations from the present data show that, for dogs to form BNA tumours through exposure to a relatively high dose level of PBNA, the period of daily dosing would occupy, or even exceed, the life-span of the species.

With regard to the situation in man, Scott (1962) stated that no tumours had been reported amongst operatives engaged in the large-scale manufacture of PBNA in a number of countries over many years. More recently, epidemiological studies (Veys, 1973; Fox, Lindars and Owen, 1974) indicate that in operatives who have been involved with widespread use of the present technical grade PBNA, the incidence of bladder tumours is no greater than in the human population at large. If it is permissible to make any correlation between the PBNA-treated animals and

PBNA-exposed man, particularly in his working environment (Kummer and Tordoir, 1975) this suggests a quite unrealistic period of exposure to PBNA for BNA tumours to appear, although in the present studies the dogs seem to have been subjected to more than 10 times the dose level in terms of mg/kg body weight. Hence, we suggest that the levels of BNA produced are so low that *N*-dephenylation of PBNA *in vivo* does not give rise to carcinogenic risk.

We thank Dr Alexander Munn, formerly Divisional Medical Officer to I.C.I. Organics Division Limited, Hexagon House, Blackley, Manchester for bringing to our attention the problem of *N*-phenyl-2-naphthylamine *N*-dephenylation in mammals, and for his help and interest. We should also like to thank Dr G. T. Steel of this laboratory for a helpful discussion.

### REFERENCES

BONSER, G. M. (1943) Epithelial Tumours of the Bladder in Dogs Induced by Pure β-Naphthylamine. *J. Path. Bact.*, **55**, 1.

BONSER, G. M., CLAYSON, D. B., JULL, J. W. & PARAH, L. N. (1952) The Carcinogenic Properties of 2-Amino-1-naphthol Hydrochloride and its Parent Amine, 2-Naphthylamine. *Br. J. Cancer*, **6**, 412.

BOYLAND, E., MANSON, D. & SIMS, P. (1953) The Preparation of *o*-Aminophenyl Sulphates. *J. chem. Soc.*, 3623.

CONZELMAN, G. M. & MOULTON, J. E. (1972) Dose-response Relationships of the Bladder Tumorigen, 2-Naphthylamine: a Study in Beagle Dogs. *J. natn. Cancer Inst.*, **49**, 193.

DEICHMANN, W. B. & RADOMSKI, J. L. (1969) Carcinogenicity and Metabolism of Aromatic Amines in the Dog. *J. natn. Cancer Inst.*, **43**, 263.

DRUCKREY, H. & KÜPFMÜLLER, K. (1948) Quantitative Analyse der Krebsentstehung. *Z. Naturf.*, **3b**, 254.

FOX, A. J., LINDARS, D. C. & OWEN, R. (1974) A Survey of Occupational Cancer in the Rubber and Cablemaking Industries: Results of Five-year Analysis, 1967–1971. *Br. J. ind. Med.*, **31**, 140.

HUEPER, W. C., WILEY, F. H. & WOLFE, D. H. (1938) Experimental Production of Bladder Tumours in Dogs by Administration of beta-Naphthylamine. *J. ind. Hyg. Toxicol.*, **20**, 46.

KUMMER, R. & TORDOIR, W. F. (1975) Phenylbetanaphthylamine (PBNA), Another Carcinogenic Agent? *Tijschr. soc. Geneesk.* (*Amsterdam*), **53**, 415.

PAULSON, G. D. & PORTNOY, C. E. (1970) Sulphate Ester Conjugates: a One-step Method for Replacing the Sulphate with an Acetyl Group. *J. agric. Fd. Chem.*, **18**, 180.

RADOMSKI, J. L. & BRILL, E. (1971) The Role of *N*-oxidation Products of Aromatic Amines in the Induction of Bladder Cancer in the Dog. *Arch. Toxikol.*, **28**, 159.

SCOTT, T. S. (1962) *Carcinogenic and Chronic Toxic Hazards at Aromatic Amines.* Amsterdam, New York: Elsevier, p. 174.

VEYS, C. A. (1973) A Study of the Incidence of Bladder Tumours in Rubber Workers. M.D. Thesis, University of Liverpool.

WALKER, G. H. & HATHWAY, D. H. (1976) Synthesis of *N*-phenyl-2-[1,4,5,8-$^{14}$C]naphthylamine, *N*-phenyl-2-[8-$^{13}$C]naphthylamine and *N*-[U-$^{14}$C]-phenyl-2-naphthylamine. *J. labelled Compounds Radiopharmaceuticals*, **12**, 199.

WILLSTÄTTER, R. & KUBLI, H. (1908) Über die Reduktion von Nitroverbindungen nach der Methode von Zinin. *Ber. dt. chem. Ges.*, **41**, 1936.